# Stories of a Country Doctor

**Published:** 1891-12

**Authors:** 


STORIES OF A COUNTRY DOCTOR. By WILLIS P. KING, M. D., Member of the American Medical Association; Member and ex-President of the Missouri State Medical Association; Assistant Chief Surgeon of the Missouri Pacific Railway Company; formerly Lecturer on Diseases of Women in the Medical Department of the Missouri State University, and Professor of Diseases of Women in the Medical Department University of Kansas City; Lecturer on Orthopedic Surgery and Clinical Surgery in the University Medical College of Kansas City, etc., etc. Second edition. With illustrations by T. A. Fitzgerald. Philadelphia: A. L. Hummel, M. D. publisher. 1891. Price, $1.00, bound in cloth.

When the first edition of this book appeared, we hardly expected to be called upon to notice it again so soon. This fact is sufficient evidence that it has a place in the heart of the profession, hence the cry for more and more as soon as the supply is exhausted. We expressed our opinion of the book so freely and, we may say, so completely before, that we hardly know how to add to, and we certainly cannot retract from, our previously expressed judgment. Every physician must have some work in his library that diverts the mind, and through that means bring rest to the body. Dr. King's book affords just the sort of reading to do this, and it has now been reduced to the nominal price of one dollar, which brings it within easy reach of almost every pocket. The publisher, Dr. A. L. Hummel, of Philadelphia, has dressed it up in a neat and practical attire, leaving nothing to be desired on that score. We again commend it to the profession as a work of light literature that is seldom equaled and still more rarely excelled.

